# A treatment planning study comparing tomotherapy, volumetric modulated arc therapy, Sliding Window and proton therapy for low-risk prostate carcinoma

**DOI:** 10.1186/s13014-016-0707-6

**Published:** 2016-09-27

**Authors:** Sergiu Scobioala, Christopher Kittel, Nicolas Wissmann, Uwe Haverkamp, Mohammed Channaoui, Omar Habibeh, Khaled Elsayad, Hans Theodor Eich

**Affiliations:** Department of Radiotherapy and Radiooncology, University Hospital of Muenster, Albert-Schweitzer-Campus 1, Gebäude A1, 48149 Muenster, Germany

**Keywords:** Prostate, Helical tomotherapy, Volumetric Modulated Arc Therapy, Sliding Window, Proton treatment, Dosimetric indices

## Abstract

**Background:**

Comparing radiation treatment plans to ascertain the optimal intensity-modulated radiation technique for low-risk prostate cancer.

**Methods:**

Treatment plans for 20 randomly selected patients were generated using the same dose objectives. A dosimetric comparison was performed between various intensity-modulated techniques, including protons. All treatment plans provided conventional treatment with 79.2Gy. Dosimetric indices for the target volume and organs at risk (OAR), including homogeneity index and four conformity indices were analyzed.

**Results:**

No statistically significant differences between techniques were observed for homogeneity values. Dose distributions showed significant differences at low-to-medium doses. At doses above 50Gy all techniques revealed a steep dose gradient outside the planning target volume (PTV). Protons demonstrated superior rectum sparing at low-to-higher doses (V10-V70, *P* < .05) and bladder sparing at low-to-medium doses (V10–V30, *P* < .05). Helical tomotherapy (HT) provided superior rectum sparing compared to Sliding Window (SW) and Rapid Arc (RA) (V10–V70, *P* < .05). SW displayed superior bladder sparing compared to HT and RA (V10–V50, *P* < .05). Protons generated significantly higher femoral heads exposure and HT had superior sparing of those.

**Conclusion:**

All techniques are able to provide a homogeneous and highly conformal dose distribution. Protons demonstrated superior sparing of the rectum and bladder at a wide dose spectrum. The radiation technique itself as well as treatment planning algorithms result in different OAR sparing between HT, SW and RA, with superior rectum sparing by HT and superior bladder sparing by SW. Radiation plans can be further optimized by individual modification of dose objectives dependent on treatment plan strategy.

**Electronic supplementary material:**

The online version of this article (doi:10.1186/s13014-016-0707-6) contains supplementary material, which is available to authorized users.

## Background

Different radiotherapy techniques and fractionation regimes are currently used for the treatment of localized prostate cancer. Conventionally fractionated intensity modulated radiotherapy (IMRT) is the most frequently applied treatment modality for prostate cancer [1–4]. IMRT can be performed either by static (“step-and-shoot” (SS), Sliding Window (SW)) or rotational approaches (helical tomotherapy (HT) or Volumetric Modulated Arc Therapy (Rapid Arc, RA). Intensity modulated proton therapy (IMPT) is also a well-known treatment modality for localized prostate cancer and is more likely to spare the organs at risk (OAR) due to steep dose gradients outside of the Bragg peak [5–9]. All of these techniques are capable of achieving treatment plans with high conformity, reducing the dose delivered to the surrounding healthy tissue and, consequently, treatment-related toxicities, especially the risk of secondary malignancy. To date there is no consensus on the “optimal overall” treatment mode [10, 11]. In this study, a statistical analysis of dosimetric parameters between HT, SW, RA and IMPT providing conventionally fractionated radiotherapy was evaluated. Similar dose objectives for PTV and OAR were used in the radiation planning for all techniques. The dosimetric indices were assessed according to the criteria of the International Commission on Radiation Units and Measurements (ICRU) Report 83 [12].

## Material and methods

Treatment plans were generated for 20 randomly selected patients with low-risk prostate cancer who received definitive HT. The databases of thin-cut 3 mm CT scans were fused with those of 1.5 T MRI scans. The target dose was calculated to be 79.2Gy. According to ICRU Report 83, at least 95 % of the prescribed dose should cover 98 % of the prostate volume (D98 %) and a maximum of 107 % of the prescribed dose should cover 2 % of the prostate volume (D2 %) [12]. Both dose objectives for PTV and OAR, as well as multiple specific physical planning support structures used for treatment planning are presented in a “Additional file 1”. Seven-field IMRT plans using the SW technique were created on the Eclipse™ 10 treatment planning system [Varian Medical Systems, Palo Alto USA]. All plans were generated for the Varian True Beam linear accelerator (LINAC) using beam energies of 15MV photons and beam angles of 0, 51, 102, 153, 204, 255 and 306°. The RA plans were created using the same treatment planning system as for SW. Two incomplete arcs from 200 to 160°, intended to protect the posterior rectum wall, were used. The plans were created using 15MV photons with a 0.5 cm leaf width. A maximum dose rate of 600 MU/min and MLC motion of 2.5 cm/s were applied. The MLC-leakage was at 1.8 %. The HT plans were assessed using Tomo Planning System Version 5 (Accuray® Inc., Sunnyvale, USA). The plans were created for the High Art HDII HT system that uses a helical slice 6MV photon beam modulated by 64 binary multileaf collimators. The IMPT plans were generated using the Eclipse™ 10 treatment planning system (Varian Medical Systems, Palo Alto USA). Each plan consisted of two opposing right and left lateral beams (gantry angles of 90° and 270°) with incident proton beam energies in the 100–235 MeV range. To create homogeneous target coverage, a multi-field optimization was used with minimum spot MUs of 0.4.

The clinical target volume (CTV) and planning target volume (PTV) were defined according to the Radiation Therapy Oncology Group (RTOG) 0521 protocol [13]. The CTV included the prostate and did not involve the seminal vesicles. A 3 mm margin in the dorsal direction and 5 mm margin in all other directions was added to delineate the PTV. The dose-volume objectives met the criteria for rectum and urinary bladder set out by the Quantitative Analyses of Normal Tissue Effects in the Clinic (QUANTEC) reports and for femoral heads established by the RTOG Prostate Group Consensus 2009 (Table 1) [13–15]. The following dosimetric indices were assessed: V10, V30, V50, V70 (defined as the percentage of organ volume receiving given radiation dose), Dmean, Dmax and D1cc for rectum and bladder, as well as V50 and D1ccm for femoral heads. A comparison of each patient was made using a scoring system that compared D2 %, D98 % and V95 values. The indices for the 20 patients were averaged to assess the advantages and disadvantages in PTV coverage and OAR sparing of the various radiation techniques.

**Table 1:** Organ at risk dose objectives

Organ	Dose	Volume (in % or in ccm as absolute volume for femoral heads)
Rectum [14]	10Gy	–^a^
	30Gy	<60 %
	50Gy	<50 %
	60Gy	<35 %
	65Gy	<25 %
	70Gy	<20 %
	75Gy	<15 %
Urinary bladder [15]	10Gy	–^a^
	30Gy	–^a^
	50Gy	<60 %
	65Gy	<50 %
	70Gy	<35 %
	75Gy	<25 %
Femoral heads [13]	50Gy	<5 %
	40Gy	≤1ccm

Abbreviations: ^a^-the dose-volume limit is not defined in the QUANTEC reports

Based on the recommendations of the ICRU 83 reports and several clinical studies, specific conformal indices were used to describe the dose distribution [12, 16]. Thus, the homogeneity index (HI) and four conformity indices (CI; these include the ICRU conformity index (CI_ICRU_), coverage index (CΔ), coverage index COV (CΔ_COV_), and conformation number (CN)) were calculated (Table 2). These indices refer to the PTV volume, partial PTV volume covered by 95 % of prescribed isodose (PTV_*pi*_), and volume treated by 95 % of prescribed isodose (TV_*PI*_) [16]. The HI characterizes dose homogeneity inside the PTV and is calculated using the near maximum (D2 %), near minimum (D98 %), and the mean dose (Dmean): D2 %–D98 % / Dmean (optimal at 0) [12]. The CN provides complementary information about the irradiation of PTV and healthy tissues, and is calculated as PTV_*pi*_*/* PTV x PTV_*pi*_*/* TV_*PI*_; a value of 1 indicates optimal result [17]. The CI_ICRU_ was described in the ICRU 62 report and is defined as the quotient TV_*PI*_*/* PTV (optimal at 1) [18]. The CΔ quantifies the radiation exposure of the surrounding healthy tissue and is defined as TV_*PI*_-PTV_*pi*_*/* PTV (optimal at 0) [19]. The CΔ_COV_ describes the coverage of the target volume by the prescribed isodose and is calculated as PTV_*pi*_*/* PTV; ideal PTV coverage is indicated by a value of 1 [20].

**Table 2:** Averaged dosimetric values for PTV and conformal indexes

	HT Mean ± SD	SW Mean ± SD	RA Mean ± SD	Protons Mean ± SD	Pairs with statistically significant differences (*P* = . < 05)/Technique with best result (mean values)
PTV					
PTV coverage (%)	98.2 ± 1.4	98.6 ± 0.8	98.3 ± 0.8	99.6 ± 0.2	Pr > HT, Pr > SW, Pr > RA / Pr > SW > RA > HT
D2 % (%)	103.3 ± 0.5	102.5 ± 0.4	102.4 ± 0.3	102.5 ± 0.4	RA > HT, SW > HT, Pr > HT / RA > SW = Pr > HT
D98 % (%)	95.3 ± 1.9	95.5 ± 0.6	95.3 ± 0.6	96.7 ± 0.4	Pr > HT, Pr > SW, Pr > RA / Pr > SW > RA > HT
Homogeneity index, HI	0.08 ± 0.02	0.07 ± 0.01	0.07 ± 0.01	0.06 ± 0.01	Pr > HT, Pr > SW, Pr > RA / Pr > SW = RA > HT
Conformation number, CN	0.81 ± 0.04	0.89 ± 0.02	0.91 ± 0.03	0.88 ± 0.02	SW > HT, RA > HT, RA > SW, RA > Pr, Pr > HT / RA > SW > Pr > HT
ICRU Conformity index, CI_ICRU_	1.20 ± 0.1	1.09 ± 0.03	1.07 ± 0.04	1.13 ± 0.03	SW > HT, SW > Pr, RA > HT, RA > Pr, Pr > HT / RA > SW > Pr > HT
Coverage index, CΔ	0.22 ± 0.1	0.11 ± 0.03	0.08 ± 0.03	0.13 ± 0.03	RA > HT, RA > SW, RA > Pr, SW > HT / RA > SW > Pr > HT
Coverage index COV, CΔ_COV_	0.98 ± 0.01	0.99 ± 0.01	0.98 ± 0.01	1.00 ± 0.0	Pr > HT, Pr > SW, Pr > RA / Pr > SW > RA = HT

*Abbreviations*: *HT* helical tomotherapy; *SW* Sliding Window; *RA* Rapid Arc; *Pr* Protons; *SD* standard deviation; *>* dosimetric superiority

A comparison of monitor units and treatment times could not be performed because the current implementation of monitor unit calculation in Eclipse Proton Planning is based on theoretical ideas and has not yet been verified by measurements due to a lack of experimental data (Proton Algorithm Reference Guide, Eclipse™ August 2013, Varian Medical Systems, Inc., Palo Alto).

In the statistical analyses, a non-parametric Wilcoxon signed-rank test was used to assess the differences between two techniques. The threshold for statistical significance was defined as *P* < .05. All statistical analyses were performed using the SPSS software (IBM SPSS Statistics 22.0).

Treatment plans were created separately by three physicists.

## Results

The dosimetric values for PTV and OAR were assessed from the patient-averaged dose-volume histogram (DVH) and are presented in detail in Tables 2 and 3. The D98 % and D2 % did not significantly vary between the techniques. However, the HT showed inferior dose distribution within the PTV, with the largest interval between D98 % and D2 % values compared to SW, RA and IMPT. The IMPT provided significantly superior homogeneity values compared to HT, SW and RA (Table 2). Comparison of the four CI, which describe the dose distribution within the PTV and the healthy tissue, demonstrated HT showed a tendency toward inferior conformation (Table 2). A paired comparison analysis showed significantly improved CN for RA compared to HT (*P* = .001), SW (*P* = .005), and IMPT (*P* = .001). The IMPT demonstrated superior CN compared to HT (*P* = .001). The CI_ICRU_ value of 1.20 obtained by HT reflects a larger coverage volume by the treatment dose than the PTV itself. For RA was found significantly superior CI_ICRU_ in contrast to HT and IMPT (*P* = .001), and SW showed superior CI_ICRU_ compared to HT (*P* = .001) and IMPT (*P* = .002). The CΔ revealed that RA was significantly superior to all other techniques, and that SW was superior to HT and IMPT (*P* < .05). IMPT showed significantly better CI_COV_ values than other modalities (*P* < .05) (Table 2).

**Table 3:** Averaged dosimetric values for organs at risk

	HT Mean ± SD	SW Mean ± SD	RA Mean ± SD	Protons Mean ± SD	Pairs with statistically significant differences (*P* = . < 05)/Technique with best result (mean values)
Urinary Bladder					
V10 (%)	42.0 ± 23.8	39.3 ± 23.3	46.8 ± 23.2	30.2 ± 18.5	Pr > HT, Pr > SW, Pr > RA, SW > HT, SW > RA / Pr > SW > HT > RA
V30 (%)	26.2 ± 18.6	23.5 ± 16.4	27.0 ± 19.1	21.2 ± 14.7	Pr > HT, Pr > SW, Pr > RA, SW > HT, SW > RA / Pr > SW > HT > RA
V50 (%)	16.8 ± 12.5	15.6 ± 11.6	17.0 ± 13.3	15.3 ± 11.5	SW > HT, SW > RA, Pr > RA, Pr > HT / Pr > SW > HT > RA
V70 (%)	6.0 ± 4.9	5.4 ± 4.8	6.4 ± 4.8	5.3 ± 4.7	Pr > SW, Pr > RA, Pr > HT / Pr > SW > HT > RA
Dmean (Gy)	17.4 ± 10.1	15.8 ± 9.6	17.9 ± 10.1	13.3 ± 9.1	Pr > HT, Pr > SW, Pr > RA, SW > HT, SW > RA / Pr > SW > HT > RA
Dmax (Gy)	82.5 ± 2.8	80.0 ± 2.8	80.9 ± 3.1	80.9 ± 1.7	SW > HT, RA > HT, Pr > HT / SW > Pr > RA > HT
D1ccm (Gy)	76.3 ± 8.2	76.2 ± 6.3	76.3 ± 8.1	76.2 ± 6.0	SW > HT, Pr > HT / Pr > SW > RA > HT
Rectum					
V10 (%)	59.1 ± 17.9	62.7 ± 16.6	69.0 ± 18.7	24.6 ± 9.2	Pr > HT, Pr > SW, Pr > RA, HT > SW, HT > RA, SW > RA / Pr > HT > SW > RA
V30 (%)	24.8 ± 7.6	32.8 ± 5.7	41.7 ± 11.3	15.7 ± 5.8	Pr > HT, Pr > SW, Pr > RA, HT > SW, HT > RA, SW > RA / Pr > HT > SW > RA
V50 (%)	12.8 ± 4.0	17.3 ± 2.5	23.5 ± 7.5	9.3 ± 3.8	Pr > HT, Pr > SW, Pr > RA, HT > SW, HT > RA, SW > RA / Pr > HT > SW > RA
V70 (%)	1.1 ± 0.7	1.5 ± 0.8	3.4 ± 1.8	1.0 ± 0.8	Pr > RA, Pr > SW, HT > SW, HT > RA, SW > RA / Pr > HT > SW > RA
Dmean (Gy)	17.3 ± 3.9	20.3 ± 3.4	24.5 ± 5.3	9.0 ± 3.4	Pr > HT, Pr > SW, Pr > RA, HT > SW, HT > RA, SW > RA / Pr > HT > SW > RA
Dmax (Gy)	78.3 ± 1.8	75.9 ± 1.2	77.3 ± 1.5	76.4 ± 2.2	Pr > HT, Pr > RA, SW > HT, SW > RA, SW > Pr / SW > Pr > RA > HT
D1ccm (Gy)	67.5 ± 4.1	68.9 ± 2.4	71.5 ± 2.5	65.9 ± 4.2	HT > SW, HT > RA, SW > RA, Pr > HT, Pr > SW, Pr > RA / Pr > HT > SW > RA
Femoral head, right					
D1ccm (Gy)	18.0 ± 2.9	32.0 ± 6.2	28.1 ± 5.3	35.2 ± 3.9	HT > SW, HT > RA, HT > Pr, RA > SW, RA > Pr / HT > RA > SW > Pr
Femoral head, left					
D1ccm (Gy)	18.1 ± 2.9	31.9 ± 5.1	26.6 ± 5.0	35.5 ± 2.5	HT > SW, HT > RA, HT > Pr, RA > Pr, RA > SW / HT > RA > SW > Pr

*Abbreviations*: *HT* helical tomotherapy; *SW* Sliding Window; *RA* Rapid Arc; *Pr* Protons; *PTV* planning target volume; *SD* standard deviation; *Dx (Gy)* dose (Gy) absorbed by certain percentage (%) or absolute volume (ccm) of the contoured structure; *Vx* percentage of organ volume exposed to certain radiation dose; *>* dosimetric superiority

**Fig. 1:**
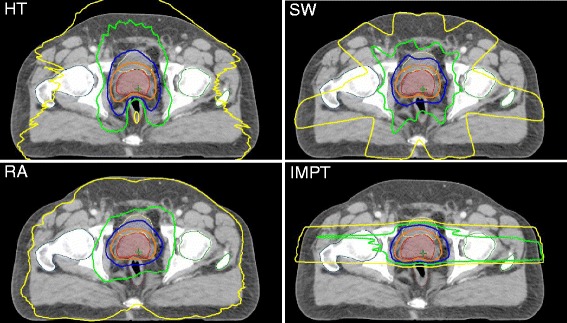
Treatment plans for tomotherapy (HT), Sliding Window (SW), Rapid Arc (RA), and protons (IMPT). Representative dose distribution with V10 (yellow), V30 (green), V50 (blue), and V70 (orange) isodoses. The prostate is delineated in red, rectum in brown, urinary bladder in yellow

**Fig. 2:**
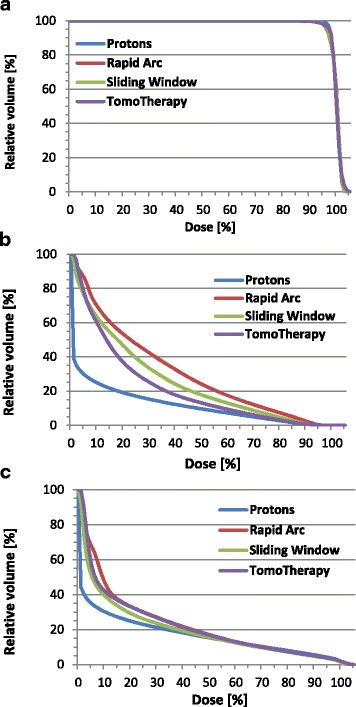
Patient-averaged dose-volume histograms. Dose-volume relationship of the planning target volume (**a**), rectal wall (**b**), and bladder wall (**c**) in the treatment plans of various IMRT techniques

**Fig. 3:**
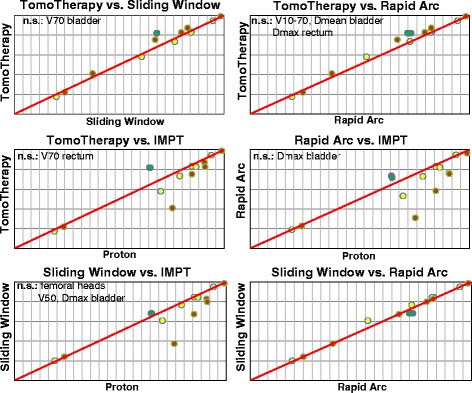
Pair-wise statistical dosimetric comparison between radiation techniques. The differences in OAR sparing are shown at dose ranges V10–V70, Dmean and Dmax for rectum and urinary bladder, and D1ccm for femoral heads. The urinary bladder is indicated in yellow, rectum in brown, femoral heads in green

A greater difference in dose distribution between the techniques was found at the low-to-medium dose ranges compared to the higher doses (Figs. 1 and 2). The patient-averaged DVH revealed IMPT had statistically superior rectum sparing at low-to-higher doses compared to all other techniques (V10–V70, *P* = <.05), with the exception of HT at V70 (Fig. 3, Table 3). Significantly lower rectum exposure was provided by HT compared to SW and RA at low-to-higher doses (V10–V70, *P* < .05) (Fig. 3, Table 3). A statistical difference in Dmean values was found in all tested pairs, with lower absolute values for protons and a maximum of absolute values for RA. Lowest Dmax value was achieved by SW, and D1ccm values revealed protons produced the lowest radiation exposure and RA produced the greatest coverage (Table 3).

The whole urinary bladder also experienced the best shielding using IMPT at lower-to-higher doses (V10–V70, *P* < .05) (Fig. 3, Table 3). SW demonstrated significantly lower bladder exposure in a wide dose spectrum than HT and RA (V10–V50, *P* < .05) (Fig. 3, Table 3). Dmean values were significantly different between all tested pairs, except for HT/RA, with lower absolute values for IMPT and higher absolute values for RA. Significantly lower Dmax values were gained for SW. D1ccm values revealed nearly the same bladder exposure for all techniques (Table 3).

D1ccm values indicated femoral heads experienced the highest exposure from protons and superior sparing from HT (Table 3). V50 revealed femoral heads received very small coverage volume (<1 %) for all techniques.

## Discussion

This study performed a statistical dosimetric comparison between different IMRT techniques using conventional fractionation to treat low-risk prostate cancer. IMRT was found to provide a conformal dose distribution, allowing dose escalation in the prostate and inferior OAR toxicity compared to 3-D conformal radiotherapy (3-D CRT) [21–24]. A dosimetric comparison between different IMRT techniques has already been performed in several series of studies [1–4, 25–32]. Tsai et al. found that HT provided superior conformity and OAR sparing compared to VMAT and static IMRT [2]. Hermann et al. demonstrated comparable homogeneity, CN values and OAR dosimetric values between VMAT, SS and SW [26]. The dosimetric comparison between static and rotational IMRT in this trial revealed superior rectum sparing by HT and superior bladder sparing by SW at low-to-higher doses (Table 3, Fig. 3). However, no accentuated priority for rectum sparing in HT plans or for bladder sparing in SW plans was made by the plans’ assessment. In our opinion, the Tomo Planning System is more sensitive to dose constraints than the Eclipse planning system. This increased sensitivity can result in greater rectum sparing in HT plans while using the same dose objectives in the Eclipse planning system for SW and RA. In addition, the high sensitivity of the Tomo Planning System can result in larger SD values for homogeneity and the conformity indices in HT plans compared to other IMRT techniques, as shown in Table 2. A higher radiation exposure for OAR at low doses was found for RA compared to HT and SW. Despite the use of two incomplete arcs intended to protect the rectum, RA demonstrated greater rectum exposure in a wide dose spectrum (Table 3). However, most of the conformity values were statistically superior for RA compared to SW and HT. Thus, assuming same dose objectives for treatment planning, differences in dose distribution within PTV and OAR can be explained both by impact of radiation technique itself and by use of different dose calculation algorithms.

Superior sparing of OAR by protons is expected due to extremely steep dose gradients outside of the Bragg peak. As demonstrated by Vargas et al. for localized prostate carcinoma, protons provided improved rectum sparing at 10–80Gy and bladder sparing at 10–35Gy compared to the IMRT techniques [5]. On the contrary, a comparable sparing of rectum, bladder and femoral heads for IMPT and VMAT techniques was observed by Georg et al. [6]. In agreement with the results found by Schwarz et al., IMPT revealed similar homogeneity values and significantly inferior conformity values compared to HT. Rectum exposure for both techniques above 65Gy was equivalent, and superior sparing of bladder and penile bulb was obtained for protons in a wide dose spectrum [7]. A recent SEER-Medicare based study observed a lower rate of gastrointestinal toxicity by IMRT-treated patients compared to proton-treated patients, and similar outcomes were observed for urinary toxicity and erectile dysfunction [9]. Based on normal tissue complication probability (NTCP) estimates, Schwarz et al. demonstrated very similar probabilities of late gastrointestinal complications for HT and IMPT [7]. Thus, the current data do not permit a definitive conclusion about the dosimetric superiority and therapy tolerance of protons compared to IMRT. In this study, IMPT showed an improved homogeneity value compared to the other techniques, and lower conformity values compared to SW and RA (Table 2), although the planning software did not allow for a robust optimization of the proton therapy. In accordance with the findings of Vargas et al., the protons demonstrated statistically superior rectum and bladder sparing at low-to-higher doses (Table 3, Fig. 3). In the case of highly curative low-risk prostate carcinoma, it is critical both to perform homogeneous prostate coverage in order to achieve a long-term local tumor control, and to minimize the risk of chronic toxicities and secondary malignances. The steep dose gradient to the rectum and bladder, even at the lowest doses, leads to the assumption of decreased risk of secondary malignancy after proton treatment. However, the clinical relevance of the higher exposure of the femoral heads through the opposing right and left lateral beams used by protons is unknown. Thus, the incidence of late toxicity, including secondary malignancy, after proton treatment should be further evaluated in long-term trials.

The dose objectives for the rectum and urinary bladder met the criteria laid out by the QUANTEC reports [14, 15]. Current dose constraints are primarily generated from 3-D CRT datasets [22]. The advanced IMRT techniques, including protons, all provide highly conformal dose distribution, performing superior OAR sparing than 3-D CRT. For this reason, dose objectives should be derived from the datasets of these advanced radiotherapy technologies.

For each radiation technique, a corresponding planning system is used to determinate dose calculation algorithms. Moreover, radiation plans can be optimized by individual modification of dose objectives for each treatment case. These arguments can diminish the relevance of the obtained results, despite the use of similar dose objectives in radiation planning. Thus, the selection criteria for dosimetric comparison of treatment plans should be further optimized.

De Meerleer et al. demonstrated that static IMRT plans had a superior ratio of tumor control probability (TCP)/normal tissue complication probabilities (NTCP) compared to 3-D CRT plans. As a result, IMRT techniques provide improved tumor control without a corresponding increase in radiation toxicities [33]. The clinical relevance of dosimetric differences between various advanced IMRT techniques should be further analyzed in relation to NTCP parameters, including risk of secondary malignancy, as already described by Hall in several reviews [34, 35].

And finally, late-responding organs that have a low α/β ratio such as the prostate are more sensitive to high fraction doses [36, 37]. Several retrospective studies demonstrated the dosimetric feasibility, clinical outcomes, and local tumor control of hypofractionated radiotherapy in the treatment of localized prostate carcinoma [38–41]. Our ongoing research will focus on evaluating dose objectives for various advanced radiation techniques, including the Cyber Knife technique, in order to simultaneously provide homogeneous prostate coverage and conformal dose distribution through the delivery of a large fraction dose.

## Conclusion

The major findings to emerge from this study are as follows: (i) All techniques revealed a homogeneous and high conformal dose distribution with more explicit differences at the low-to-medium dose ranges. At the dose above 50Gy all techniques demonstrated a steep dose gradient outside the PTV, implying a similar frequency and severity of acute toxicities. (ii) When using the same dose objectives during planning, IMPT revealed superior rectum and bladder sparing in a wide dose spectrum. This trend was more relevant at the low-to-medium doses. The superior OAR sparing by IMPT was concordant with results of Vargas et al, while did not match the results of Georg et al [5, 6]. In contrast to findings of Schwarz et al., IMPT revealed conformity values comparable to SW and RA techniques and even superior to HT [7]. (iii) While the slight advantage in bladder sparing by SW can be explained by the use of different dose calculation algorithms during the treatment plan optimization process, the highly significant superior rectum sparring by HT can only be partially explained by the algorithm and is assumed to be a result of the HT treatment technique. Of course, the radiation plans can be further optimized by individual modification of dose objectives in dependence of treatment plan strategy.

## Additional file

Additional file 1: Support strukture and dose objectives used for OAR. (DOCX 15 kb)

## Abbreviations

3-D CRT: 3-dimensional conformal radiotherapy
CI_ICRU_: ICRU conformity index
CN: conformation number
CT: computed tomography
CTV: clinical target volume
CΔ: coverage index
CΔ_COV_: coverage index COV
DVH: dose-volume histogram
HI: homogeneity index
HT: helical tomotherapy
ICRU: International Commission on Radiation Units and Measurements
IMPT: intensity modulated proton therapy
IMRT: intensity modulated radiotherapy
LINAC: linear accelerator
MLC: multileaf collimator
MRI: magnetic resonance imaging
NTCP: normal tissue complication probabilities
OAR: organ at risk
PTV: planning target volume
QUANTEC: Quantitative Analyses of Normal Tissue Effects in the Clinic
RA: Rapid Arc
RTOG: Radiation Therapy Oncology Group
SW: Sliding Window
TCP: tumor control probability
VMAT: Volumetric Modulated Arc Therapy

## References

[1] Al-Mamgani A, Heemsbergen WD, Peeters ST, Lebesque JV (2009). Role of intensity-modulated radiotherapy in reducing toxicity in dose escalation for localized prostate cancer. Int J Radiat Oncol Biol Phys.

[2] Tsai CL, Wu JK, Chao HL, Tsai YC, Cheng JC (2011). Treatment and dosimetric advantages between VMAT, IMRT, and helical tomotherapy in prostate cancer. Med Dosim.

[3] Davidson MT, Blake SJ, Batchelar DL, Cheung P, Mah K (2011). Assessing the role of volumetric modulated arc therapy (VMAT) relative to IMRT and helical tomotherapy in the management of localized, locally advanced, and post-operative prostate cancer. Int J Radiat Oncol Biol Phys.

[4] Wolff D, Stieler F, Welzel G, Lorenz F, Abo-Madyan Y, Mai S (2009). Volumetric modulated arc therapy (VMAT) vs. serial tomotherapy, step-and-shoot IMRT and 3D-conformal RT for treatment of prostate cancer. Radiother Oncol.

[5] Vargas C, Fryer A, Mahajan C, Indelicato D, Horne D, Chellini A (2008). Dose-volume comparison of proton therapy and intensity-modulated radiotherapy for prostate cancer. Int J Radiat Oncol Biol Phys.

[6] Georg D, Hopfgartner J, Gòra J, Kuess P, Kragl G, Berger D (2014). Dosimetric considerations to determine the optimal technique for localized prostate cancer among external photon, proton, or carbon-ion therapy and high-dose-rate or low-dose-rate brachytherapy. Int J Radiat Oncol Biol Phys.

[7] Schwarz M, Pierelli A, Fiorino C, Fellin F, Cattaneo GM, Cozzarini C (2011). Helical tomotherapy and intensity modulated proton therapy in the treatment of early stage prostate cancer: a treatment planning comparison. Radiother Oncol.

[8] Widesott L, Pierelli A, Fiorino C, Lomax AJ, Amichetti M, Cozzarini C (2011). Helical tomotherapy vs. intensity-modulated proton therapy for whole pelvis irradiation in high-risk prostate cancer patients: dosimetric, normal tissue complication probability, and generalized equivalent uniform dose analysis. Int J Radiat Oncol Biol Phys.

[9] Sheets NC, Goldin GH, Meyer AM, Wu Y, Chang Y, Stürmer T (2012). Intensity-modulated radiation therapy, proton therapy, or conformal radiation therapy and morbidity and disease control in localized prostate cancer. JAMA.

[10] de Crevoisier R, Castelli J, Guérif S, Pommier P, Créhange G, Chauvet B, Lagrange JL (2014). Prostate cancer: what treatment techniques for which tumors? Ethical and methodological issues. Cancer Radiother.

[11] Khadige M, Peiffert D, Supiot S (2014). What is the level of evidence of new techniques in prostate cancer radiotherapy?. Cancer Radiother.

[12] ICRU, Report 83 (2010). Prescribing, recording, and reporting Intensity-Modulated Photon-Beam.

[13] http://www.cancer.gov/clinicaltrials/search/view?cdrid=462375&version=healthprofessional#ContactInfo_CDR0000462375. Accessed 1 Oct 2011.

[14] Michalski JM, Gay H, Jackson A, Tucker SL, Deasy JO (2010). Radiation dose-volume effects in radiation-induced rectal injury. Int J Radiat Oncol Biol Phys.

[15] Viswanathan AN, Yorke ED, Marks LB, Eifel PJ, Shipley WU (2010). Radiation dose-volume effects of the urinary bladder. Int J Radiat Oncol Biol Phys.

[16] Haverkamp U, Norkus D, Kriz J, Müller Minai M, Prott FJ, Eich HT (2014). Optimization by visualization of indices. Strahlenther Onkol.

[17] Van’t Riet A, Mak AC, Moerland MA, Elders LH, van der Zee W (1997). A conformation number to quantify the degree of conformality in brachytherapy and external beam irradiation: application to the prostate. Int J Radiat Oncol Biol Phys.

[18] ICRU, Report 62 (1999). Prescribing, recording and reporting photon beam therapy (Supplement to ICRU Report 50).

[19] Van Gellekom MP, Moerland MA, Battermann JJ, Lagendijk JJ (2004). MRI-guided prostate brachytherapy with single needle method--a planning study. Radiother Oncol.

[20] Das IJ, Cheng CW, Healey GA (1995). Optimum field size and choice of isodose lines in electron beam treatment. Int J Radiat Oncol Biol Phys.

[21] Wang-Chesebro A, Xia P, Coleman J, Akazawa C, Roach M (2006). Intensity-modulated radiotherapy improves lymph node coverage and dose to critical structures compared with three-dimensional conformal radiation therapy in clinically localized prostate cancer. Int J Radiat Oncol Biol Phys.

[22] Pollack A, Zagars GK, Starkschall G, Antolak JA, Lee JJ, Huang E (2002). Prostate cancer radiation dose response: results of the M. D. Anderson phase III randomized trial. Int J Radiat Oncol Biol Phys.

[23] Pollack A, Zagars GK, Starkschall G, Childress CH, Kopplin S, Boyer AL, Rosen II (1996). Conventional vs. conformal radiotherapy for prostate cabcer: preliminary results of dosimetry and acute toxicity. Int J Radiat Oncol Biol Phys.

[24] Zietman AL, DeSilvio ML, Slater JD, Rossi CJ, Miller DW, Adams JA, Shipley WU (2005). Comparison of conventional dose vs. high-dose conformal radiation therapy in clinically localized adenocarcinoma of the prostate: a randomized controlled trial. JAMA.

[25] Pasquier D, Cavillon F, Lacornerie T, Touzeau C, Tresch E, Lartigau E (2013). A dosimetric comparison of tomotherapy and volumetric modulated arc therapy in the treatment of high-risk prostate cancer with pelvic nodal radiation therapy. Int J Radiat Oncol Biol Phys.

[26] Herman Tde L, Schnell E, Young J, Hildebrand K, Algan O, Syzek E (2013). Dosimetric comparison between IMRT delivery modes: step-and-shoot, sliding window, and volumetric modulated arc therapy - for whole pelvis radiation therapy of intermediate-to-high risk prostate adenocarcinoma. J Med Phys.

[27] Cao D, Holmes TW, Afghan MK, Shepard DM (2007). Comparison of plan quality provided by intensity-modulated arc therapy and helical tomotherapy. Int J Radiat Oncol Biol Phys.

[28] Rao M, Yang W, Chen F, Sheng K, Ye J, Mehta V (2010). Comparison of Elekta VMAT with helical tomotherapy and fixed field IMRT: plan quality, delivery efficiency and accuracy. Med Phys.

[29] Rong Y, Tang G, Welsh JS, Mohiuddin MM, Paliwal B, Yu CX (2011). Helical tomotherapy versus single-arc intensity-modulated arc therapy: a collaborative dosimetric comparison between two institutions. Int J Radiat Oncol Biol Phys.

[30] Palma D, Vollans E, James K, Nakano S, Moiseenko V, Shaffer R (2008). Volumetric modulated arc therapy for delivery of prostate radiotherapy: comparison with intensity-modulated radiotherapy and three-dimensional conformal radiotherapy. Int J Radiat Oncol Biol Phys.

[31] Kopp RW, Duff M, Catalfamo F, Shah D, Rajecki M, Ahmad K (2011). VMAT vs. 7-field IMRT: assessing the dosimetric parameters of prostate cancer treatment with a 292-patient sample. Med Dosim.

[32] Iori M, Cattaneo GM, Cagni E, Fiorino C, Borasi G, Riccardo C (2008). Dose-volume and biological-model based comparison between helical tomotherapy and (inverse-planned) IMAT for prostate tumours. Radiother Oncol.

[33] De Meerleer GO, Vakaet LA, De Gersem WR, De Wagter C, De Naeyer B, De Neve W (2000). Radiotherapy of prostate cancer with or without intensity modulated beams: a planning comparison. Int J Radiat Oncol Biol Phys.

[34] Hall E (2006). Intensity-modulated radiation therapy, protons, and the risk of second cancers. Int J Radiat Oncol Biol Phys.

[35] Hall EJ, Wuu CS (2003). Radiation-induced second cancers: the impact of 3D-CRT and IMRT. Int J Radiat Oncol Biol Phys.

[36] Fowler J, Chappell R, Ritter M (2001). Is alpha/beta for prostate tumors really low?. Int J Radiat Oncol Biol Phys.

[37] Wang JZ, Guerrero M, Li XA (2003). How low is the alpha/beta ratio for prostate cancer?. Int J Radiat Oncol Biol Phys.

[38] Martin JM, Rosewall T, Bayley A, Bristow R, Chung P, Crook J (2007). Phase II trial of hypofractionated image-guided intensity-modulated radiotherapy for localized prostate adenocarcinoma. Int J Radiat Oncol Biol Phys.

[39] Arcangeli G, Fowler J, Gomellini S, Arcangeli S, Saracino B, Petrongari MG (2011). Acute and late toxicity in a randomized trial of conventional versus hypofractionated three-dimensional conformal radiotherapy for prostate cancer. Int J Radiat Oncol Biol Phys.

[40] King CR, Brooks JD, Gill H, Presti JC (2012). Long-term outcomes from a prospective trial of stereotactic body radiotherapy for low-risk prostate cancer. Int J Radiat Oncol Biol Phys.

[41] McBride SM, Wong DS, Dombrowski JJ, Harkins B, Tapella P, Hanscom HN (2012). Hypofractionated stereotactic body radiotherapy in low-risk prostate adenocarcinoma: preliminary results of a multi-institutional phase 1 feasibility trial. Cancer.

