# Supplementary material for: A treatment planning study comparing tomotherapy, volumetric modulated arc therapy, Sliding Window and proton therapy for low-risk prostate carcinoma
Source: Radiat Oncol. 2016 Sep 27;11:128. doi: 10.1186/s13014-016-0707-6 (PMC5037612; doi:10.1186/s13014-016-0707-6)
Supplement: Additional file 1: — Support strukture and dose objectives used for OAR. (DOCX 15 kb) [file 13014_2016_707_MOESM1_ESM.docx]

| OAR | physical structure | dose objectives |
| --- | --- | --- |
|  |  | Volume [%] < Dose value [Gy] |
| Rectum | ph_rectum_s | 65 < 6 |
|  |  | 40 < 15 |
|  |  | 20 < 30 |
| Bladder | ph_bladder_s | 20-30 < 2-7* |
|  |  | 10-15 < 13-18* |
| Femoral head | Femoral head | 1 < 18 |
| Normal tissue | ph_Upper  ph_Lower | 0 < 81  50 < 60 |
|  |  | 0 < 72 |

* depending on share volume of bladder and PTV

During the optimization extra/physical structures were used for OAR sparing and to reduce the dose in the normal tissue. “Ph_rectum_s” and “ph_bladder_s” are the volume of the OAR without the volume of the OAR in the PTV. “Ph_Upper” is a PTV margin of 3cm. “Ph_Lower” is a PTV margin of 5mm up to 3cm. These two structure were used to minimize the dose in the normal tissue. No normal tissue object was used in the RA or SW plans.

The used dose objectives for PTV were as follows: 0% < 81Gy; 50% < 79.2Gy; 50% > 79.2Gy; 100% > 77.7Gy
